# The number of drones to inseminate a queen with has little potential for optimization of honeybee breeding programs

**DOI:** 10.1186/s41065-024-00332-0

**Published:** 2024-08-27

**Authors:** Manuel Du, Richard Bernstein, Andreas Hoppe

**Affiliations:** Breeding and Genetics, Institute for Bee Research Hohen Neuendorf, Friedrich-Engels-Str. 32, Hohen Neuendorf, 16540 Germany

**Keywords:** Instrumental insemination, Optimized breeding schemes, Honeybees, Inbreeding, Genetic gain

## Abstract

**Background:**

Mating control is a crucial aspect of honeybee breeding. Instrumental insemination of queens gives the breeder maximum control over the genetic origin of the involved drones. However, in addition to the drones’ descent, the breeder’s control also extends over the number of drones to use for inseminations. Thus far, this aspect has largely been ignored in attempts to optimize honeybee breeding schemes. The literature provides some comparisons between single drone inseminations (SDI) and multi drone inseminations (MDI) but it is unclear whether the number of drones used in MDI is a relevant parameter for the optimization of honeybee breeding programs.

**Methods:**

By computer simulations, we investigated the effect of the number of drones per inseminated queen in breeding programs that relied on best linear unbiased prediction (BLUP) breeding values. We covered a range of 1 to 50 drones per queen and observed the developments of genetic gain and inbreeding over a period of 20 years. Hereby, we focused on insemination schemes that take the drones for one queen from a single colony.

**Results:**

SDI strategies led to 5.46% to 14.19% higher genetic gain than MDI at the cost of 6.1% to 30.2% higher inbreeding rates. The number of drones used in MDI settings had only a negligible impact on the results. There was a slight tendency that more drones lead to lower genetic gain and lower inbreeding rates but whenever more than five drones were used for inseminations, no significant differences could be observed.

**Conclusion:**

The opportunities to optimize breeding schemes via the number of drones used in inseminations are very limited. SDI can be a viable strategy in situations where breeders are interested in genetically homogeneous offspring or precise pedigree information. However, such strategies have to account for the fact that the semen from a single drone is insufficient to fill a queen’s spermatheca, whence SDI queens will not build full-strength colonies. When deciding for MDI, breeders should focus on collecting enough semen for a succesful insemination, regardless of how many drones they need for this purpose.

## Background

Humanity has been keeping honeybees (*Apis mellifera*) since at least 2400 BCE [1, 2]. But while human management inevitably affected the genetics of honeybee populations [3], the species was never fully domesticated, presumably due to the continued mating of kept queens with wild drones [1, 4]. In the 19th century, honeybees raised the interest of Gregor Mendel (1822–1884) [5, 6], who attempted to breed superior colonies. However, rather than selecting favorable colonies of his local stock for reproduction, he aimed to hybridize bees from different origins to combine their desired properties [7, 8]. After the rediscovery of Mendel’s work in 1900 [9], theoretical and statistical advances led to a boost in animal breeding efforts [10, 11]. Consequently, breeding attempts were made to achieve genetic improvement also in honeybees by selecting the best colonies from relatively closed populations [12]. After few decades, however, it still had to be concluded that most breeding efforts in the honeybee had been futile [13]. The reason was found in the complicated mating biology, which continues to pose challenges to successful honeybee breeding programs to this day [14–16].

Honeybees separate into three castes: queens, drones, and workers, of which typically only the former two reproduce. Queens and workers are female and diploid, while the male drones develop from unfertilized eggs and are haploid. At its peak, a honeybee colony consists of a single queen, several hundreds of drones and tens of thousands of workers. The drones and workers are all offspring of the queen [4]. When a new queen emerges, a few days after hatching she will perform one or several nuptial flights on which she mates in mid-air with several drones from other colonies [17]. The sperm she collects during this flight is stored in her spermatheca and used for the rest of her life to fertilize eggs that develop into workers or daughter queens [4].

For breeders, this mating behavior comes with the difficulty to control which drones are allowed to reproduce. This is especially crucial because queens only mate once in their lives. If a queen mates with undesired drone material, she is irreversibly spoiled for the purpose of creating a new generation of queens.

First attempts to let queens and drones mate under controlled conditions date back to the 18th century [18], but it was not until the second half of the 19th century until first successes materialized. In 1868, several reports emerged announcing successful matings by artificially delaying the flight hours of queens and their intended drone mates to times of the day when other drones had already returned to their hives [19]. This method was rediscovered and popularized as ‘Horner System’ in the 21st century [20]. It is still attributed to have potential for small-scale breeding programs [21].

In the 1900s, isolated mating stations became popular in Europe [22, 23]: Colonies with the desired drones are brought to geographically secluded areas that are otherwise void of honeybees. When a virgin queen is brought to such a mating station, the only available drones for her mating are the intended ones. In large parts of Europe, isolated mating stations are still the predominant mode of mating control [24, 25]. However, the geographic and logistic requirements for isolated mating stations to provide reliable mating success are high [26] and most breeding programs struggle to find enough suitable locations.

The 1920s saw the advent of instrumental insemination of honeybee queens. Claims of successful instrumental inseminations of honeybees had already been made earlier [27], but are mostly judged as dubious [28, 29]. Watson [13] was the first to prove reproducible success. However, because the queen’s natural polyandry was still unknown at the time, he mostly used the sperm of a single drone for insemination. Only when the initial insemination had failed, he followed up with further drones. Such *single drone inseminations* (SDI) do not provide the queens with enough semen to build a full-strength colony, whence they generally only survive for several months [30]. Nevertheless, single drone inseminations are still used today, mainly in experimental setups where one is interested in genetically similar offspring of a queen or precise pedigree information [26, 31–33]. Nolan [28] reported success in inseminations of queens with multiple drones. By coincidence, the drones used in his *multi drone inseminations* (MDI) were always taken from the same colony. In the following years, further advances increased the success rates of instrumental inseminations. These included improvement of the insemination instruments [34, 35], narcotization of the queen with CO_2_ [36], and the use of diluents [37]. Today, instrumental insemination has developed into a reliable method of queen fertilization [38, 39].

By isolated mating stations or instrumental insemination, breeders can determine which queens shall reproduce via their drones. To make apt selection decisions, they need to be able to reliably assess the genetic quality of colonies. For livestock species, such genetic evaluations are facilitated by best linear unbiased prediction (BLUP) breeding value estimation derived by Henderson [40]. After adaptation to the honeybee [41], this method has yielded considerable success in terms of genetic progress [24, 42, 43]. As input parameter, BLUP requires an inverse relationship matrix between all involved colonies. Exact relationship calculations between honeybee colonies are only possible with SDI. Otherwise, for female offspring of a queen, the specific father drone is not detectable. However, if queens mate with multiple drones that all share a common grand-dam (typical situation on Central European mating stations) or even a common dam (possible for instrumental insemination), approximate relationships can be calculated based on probabilistic considerations [41, 44–46].

The more reliably the genetic background of the mating drones is known, the more accurate is the approximated relationship matrix and hence the estimated breeding values. This translates into a faster genetic progress under truncation selection if queens are inseminated with drones from a single colony in comparison to mating on mating stations, where the drones come from a sister group of drone producing queens [47]. But when queens are instrumentally inseminated, the breeder’s control extends not only over the number of colonies the drones are taken from, but also over the number of drones used in the fertilizing process. As the number of drones to mate a queen regularly occurs in the formulas for approximate relationship calculations [45, 46], the question arises, whether this parameter gives an opportunity for optimization of honeybee breeding programs. It is therefore of great interest, how the number of drones to inseminate a queen with affects genetic progress and inbreeding rates under selection based on BLUP-estimated breeding values.

Numerous studies have investigated the impact of insemination volume on the queen and her colony [48–52]. Consensus has been reached that an insemination volume of 8–12 $\mu$l is required to sufficiently fill a queen’s spermatheca to let her raise a vital colony [38, 39]. Since a single drone can provide approximately 1 $\mu$l of semen [39, 53], several authors conclude sperm to be collected from 8 to 12 drones [54, 55], whereas Cobey [56] notes that 16 to 20 drones are often necessary. In general, however, the number of drones used in an insemination is mostly seen as secondary, as long as the desired semen volume is reached. Even in studies that compare SDI and MDI, the observed differences are often attributed to the semen volume, rather than the number of drones [57, 58]. Given the dependency of approximated relationship matrices on the number of drones that fertilize a queen, it is to date unclear if this judgment is justified in the context of BLUP-based honeybee breeding.

The only theoretical quantitative genetic study concerned with the number of drones to be used for inseminations that we are aware of is a simulation study by Kistler et al. [59]. They compared honeybee breeding schemes in which queens were inseminated with one, eight or sixteen drones. However, in their study, selection was based on phenotypes instead of breeding values and was therefore not affected by calculations of approximate relationship matrices. They found that SDI led to lower genetic progress and higher inbreeding rates than MDI, while the differences between inseminations with eight or sixteen drones were negligible.

In practice, the data base www.beebreed.eu reveals that several breeders inseminate their queens with only four drones from one colony. Reportedly, they hereby try to achieve some of the advantages of SDI with slightly stronger colonies. Thus inseminated queens can produce full-size colonies but mostly survive no longer than one year. To date, however, any reports of success or failures with this strategy remain purely anecdotal and are not backed by scientific studies.

In our study, we use stochastic computer simulations to investigate the effect of the number of drones per inseminated queen in breeding programs that rely on BLUP breeding values. We cover a wide range of 1 to 50 drones per queen and investigate the developments of genetic gain and inbreeding over the period of 20 years. Hereby, we focus on insemination schemes that take the drones for one queen from a single colony. This strategy is used in several real-world breeding systems [59, 60] and has yielded promising results in previous simulation studies [47, 61].

## Material and methods

Honeybee populations comprising 500 colonies per year were simulated with the program BeeSim [62, 63]. Over the time of 20 years, these populations were selected for a single additive quantitative trait.

### Genetic model

Accounting for the fact that most economically relevant breeding traits in honeybees (like honey yield or gentleness) are commonly affected by the queen and her workers [24, 64, 65], we modeled the trait with (maternal) queen effects and (direct) worker effects. These are largely equivalent to maternal and direct effects as they also occur in other livestock species [66]. Two different sets of genetic parameters were considered. Both traits came with maternal, direct, and residual variances of $\sigma _{A,m}^2=1$, $\sigma _{A,d}^2=2$, and $\sigma _{E}^2=4$, respectively. They differed in the correlation between maternal and direct effects, which was chosen either $r_{md}=-0.18$ or $r_{md}=-0.53$. The same set of genetic parameters had previously been used in other honeybee breeding simulations [47, 61]. We write $\sigma _{A,md}$ for the covariance $\sigma _{A,m}\cdot \sigma _{A,d}\cdot r_{md}$ and denote the matrix $$\left( \begin{array}{cc}\sigma _{A,m}^2 & \sigma _{A,md}\\ \sigma _{A,md} & \sigma _{A,d}^2\end{array}\right)$$ by $\varvec{\Sigma }_A$.

Traits were simulated according to the infinitesimal model [67] with the standard honeybee specific adaptations as described e. g. in [15, 59, 62]: A base population queen *Q* obtained her maternal and direct breeding values $$\textbf{TBV}_Q=\left( \begin{array}{c}\textrm{TBV}_{Q,m}\\ \textrm{TBV}_{Q,d}\end{array}\right)$$ randomly according to a $\mathcal N(\varvec{0},\varvec{\Sigma }_A)$-distribution. Later generations inherited their true breeding values according to the following rules.

- A drone *D* with dam queen *Q* obtained the breeding value
  $$\begin{aligned} \textbf{TBV}_D=\frac{1}{2}\textbf{TBV}_Q+\varvec{\Phi }_{Q,D}. \end{aligned}$$
  Here, $\varvec{\Phi }_{Q,D}$ denotes random Mendelian sampling with distribution $\mathcal N\left(\varvec{0},\frac{1-F_Q}{4}\varvec{\Sigma }_A\right)$, $F_Q$ being *Q*’s inbreeding coefficient.
- A queen *R* with dam queen *Q* and sire drone *D* obtained the breeding value
  $$\begin{aligned} \textbf{TBV}_R=\frac{1}{2}\textbf{TBV}_Q+\varvec{\Phi }_{Q,R} + \textbf{TBV}_D. \end{aligned}$$
  Again, $\varvec{\Phi }_{Q,R}$ denotes random Mendelian sampling with distribution $\mathcal N\left(\varvec{0},\frac{1-F_Q}{4}\varvec{\Sigma }_A\right)$.
- A worker group *W* with dam queen *Q* and sire drones $D_1$, ..., $D_{N_D}$ obtained the breeding value
  $$\begin{aligned} \textbf{TBV}_W=\frac{1}{2}\textbf{TBV}_Q+\frac{1}{N_D}\sum _{i=1}^{N_D} \textbf{TBV}_{D_i}. \end{aligned}$$

These inheritance rules reflect the haploidy of drones and model the true breeding value of a worker group as the average over infinitely many workers [59, 68].

### Selection and mating

For each colony, consisting of a queen *Q* and her worker group *W*, we generated a phenotypic value as

$$\begin{aligned} \textrm{Phen}_{Q,W}=\textrm{TBV}_{Q,m}+\textrm{TBV}_{W,d}+R_{QW}, \end{aligned}$$

where the residual $R_{QW}$ was the realization of a $\mathcal N\left(0,\sigma _E^2\right)$-distributed random variable. Based on the recorded pedigrees and phenotypic values, a BLUP breeding value estimation was performed, for which the inverse relationship matrix was calculated as specified in [46]. For this breeding value estimation, colonies were randomly assigned to one of 40 apiaries. Each year, the two-year-old queens with the highest estimated breeding values were selected to produce the next generation of queens. The selection criterion for a queen consisted of the sum of the maternal and direct estimated breeding values of her worker group, because this value represents the expected genetic quality of her daughters [41, 69]. Two different values for the number of selected queens per year were covered, $N_Q=50$ and $N_Q=100$, and all selected queens produced the same number of daughters (10 in case of $N_Q=50$, 5 in case of $N_Q=100$). After hatching, queens were inseminated with drones from one of $N_S=40$ or $N_S=80$ sire colonies. The sire queens were selected among the two-or-three-year-old queens. Following common practice [70], the selection criterion for queens for this purpose was the sum of their own maternal and direct estimated breeding values. Groups of eight sire queens formed an insemination station and sister groups of queens were always fertilized at the same insemination station. It was avoided to inseminate queens with drones from their own mother or an aunt. The general reproductional setting was thus identical with setting IIS_mix_ in [47] and setting SCI in [61].

The main focus of our study lay on the number $N_D$ of drones to inseminate a queen with. We let $N_D$ cover all values from 1 to 20, as well as the additional values 25, 30, 40, and 50. The combination of two respective values for $r_{md}$ ($-0.18$, $-0.53$), $N_Q$ (50, 100), and $N_S$ (40, 80) with 24 different values for $N_D$ (1–20, 25, 30, 40, 50) led to a total of 192 simulation settings. In order to be able to detect even slight differences between the respective settings, simulations for each setting were repeated 1000 times.

## Results

### Genetic gain

**Fig. 1:**
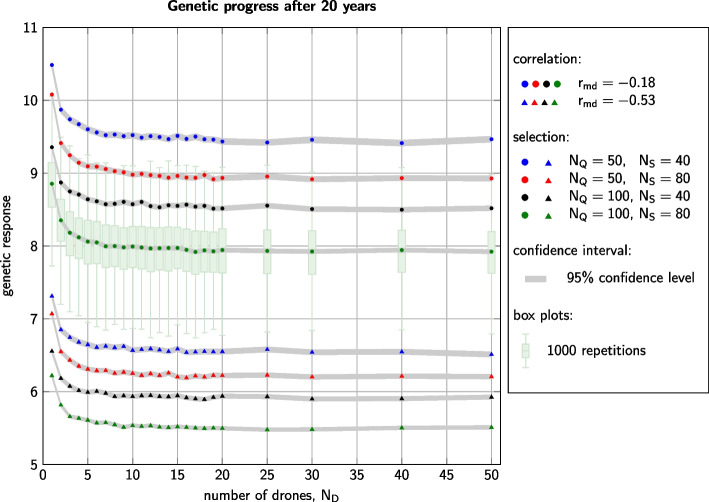
Genetic progress after 20 years of selection for all 192 scenarios. Genetic gain is measured in the performance criterion, i. e., the sum of the queen’s maternal breeding value and the worker group’s direct breeding value [68]. Dots and triangles represent the average values taken over 1000 repetitions. The gray area signifies the 95% confidence interval for these mean values. The box plots show the spread of results over the 1000 repetitions. For better visual clarity, box plots are only depicted for settings with $r_{md}=-0.18$, $N_Q=100$, and $N_S=80$ and excessive outliers are not shown. The spread of results for the other settings was similar

All 192 simulated scenarios yielded genetic progress over the course of 20 years. On average, the genetic gain in year 20 amounted to between 5.48 units ($r_{md}=-0.53$, $N_Q=100$, $N_S=80$, $N_D=25$) and 10.49 units ($r_{md}=-0.18$, $N_Q=50$, $N_S=40$, $N_D=1$). The correlation $r_{md}$ between maternal and direct effects had the greatest effect on the outcome (Fig. 1): *Ceteris paribus*, the genetic progress for $r_{md}=-0.18$ was between 42.33% and 45.37% higher than for $r_{md}=-0.53$. In comparison, the effects of selection intensity ($N_Q$ and $N_S$) were secondary but still clearly discernible. More intense selection (i. e., lower values of $N_Q$ and $N_S$) led to higher genetic gain. Hereby, halving (resp. doubling) the number $N_Q$ of dams had a greater effect than changing the number $N_S$ of sires. Due to the high number of 1000 repetitions per simulated setting, all these differences were highly significant. For any fixed number $N_D$ of drones per queen, pairwise comparison of the results for the eight different combinations of $r_{md}$, $N_Q$, and $N_S$ always yielded *p*-values below $10^{-19}$ (Welch t-test).

For each of the eight combinations of $r_{md}$, $N_Q$, and $N_S$, we observed that on average SDI yielded 5.46% to 14.19% higher genetic progress than the MDI strategies. All of these differences were highly significant with *p*-values below $10^{-62}$ (Welch t-test). Comparing the results for different levels of polyandry, i. e., for MDI with different numbers of drones, the outcomes were less clear. Up until $N_D\approx 5$, we could still detect that the addition of one further drone led to a decrease in genetic gain, which was, however, not in all cases significant. For $N_D\gtrapprox 6$, the genetic progress appeared to no longer depend on the number of drones used for insemination.

Considering the 1000 replicates for each scenario, we observed a standard deviation in the genetic gain after 20 years between 0.37 units ($r_{md}=-0.53$, $N_Q=100$, $N_S=80$, $N_D=5$) and 0.68 units ($r_{md}=-0.18$, $N_Q=50$, $N_S=40$, $N_D=1$). Relative to the achieved genetic gain, these values amounted to between 5.16% ($r_{md}=-0.18$, $N_Q=100$, $N_S=80$, $N_D=18$) and 8.81% ($r_{md}=-0.53$, $N_Q=50$, $N_S=40$, $N_D=17$).

### Inbreeding

In all 192 simulated scenarios, considerable inbreeding had built up after 20 years. Average inbreeding coefficients in year 20 lay between 0.069 ($r_{md}=-0.18$, $N_Q=100$, $N_S=80$, $N_D=25$) and 0.234 ($r_{md}=-0.53$, $N_Q=50$, $N_S=40$, $N_D=1$). In practice, the relevance of total inbreeding coefficients is limited, because they heavily depend on the depth of the recorded pedigree. Instead, one generally considers the increase of inbreeding per generation, which in our case can be calculated via the formula [47, 71]

$$\begin{aligned} \Delta F=1-(1-F_{20})^{\overline{GI}/19}. \end{aligned}$$

Here, $F_{20}$ denotes the average inbreeding coefficient in year 20 and $\overline{GI}$ is the average generation interval (so $\overline{GI}/19$ is the inverse number of generations until year 20). In our simulations, maternal generation intervals were always two years, and paternal generation intervals were two or three years, resulting in values of $\overline{GI}$ between 2.14 and 2.18 years, without clear dependence on the simulated scenarios. The resulting generational inbreeding rates amounted to between 0.85% and 3.18%.

**Fig. 2:**
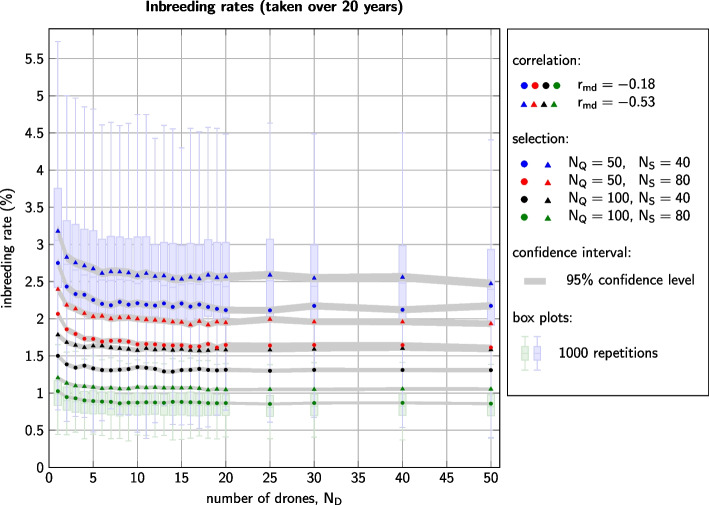
Generational inbreeding rates for all 192 scenarios. Dots and triangles represent the average values taken over 1000 repetitions. The grey area signifies the 95% confidence interval for these mean values. The box plots show the spread of results over the 1000 repetitions. Box plots are only depicted for settings with ($r_{md}=-0.18$, $N_Q=100$, $N_S=80$) and ($r_{md}=-0.53$, $N_Q=50$, $N_S=40$) to show the range of standard deviations

The parameter which had the greatest influence on inbreeding rates was the number $N_Q$ of selected queen dams (Fig. 2). With all other parameters left equal, halving the number of dams from $N_Q=100$ to $N_Q=50$ yielded an increase in inbreeding rates by between 55.9% and 101.3%. In comparison, halving the number $N_S$ of drone producers increased the inbreeding rates only by between 27.9% and 54.8%. The influence of the correlation $r_{md}$ between maternal and direct effects was lower but still clearly detectable. Inbreeding rates for $r_{md}=-0.53$ were between 13.7% and 24.9% higher than those for $r_{md}=-0.18$. All these differences were highly significant with *p*-values below $10^{-18}$ (Welch t-test).

Fixing any of the eight combinations of $r_{md}$, $N_Q$, and $N_S$, the SDI strategy led on average to 6.1% to 30.2% higher inbreeding rates than MDI breeding. All of these differences were significant with *p*-values below $10^{-5}$ (Welch t-test). As in our analysis of genetic gain, the comparison of inbreeding rates for MDI strategies with different numbers $N_D$ of drones per insemination was less conclusive. Again, for small numbers of $N_D$, up until $N_D\approx 5$, a general negative connection between $N_D$ and inbreeding rates could be detected. Note however, that in the setting defined by $r_{md}=-0.18$, $N_Q=100$, and $N_S=40$ the inbreeding rate for $N_D=4$ was (insignificantly) higher than for $N_D=3$. For higher numbers of $N_D$ we observed little to no effect of $N_D$ on the inbreeding rates.

The 1000 replications for each scenario yielded standard deviations in inbreeding rates between 0.22 percent points ($r_{md}=-0.18$, $N_Q=100$, $N_S=80$, $N_D=19$) and 1.11 percent points ($r_{md}=-0.53$, $N_Q=50$, $N_S=40$, $N_D=1$). The latter setting also yielded the highest individual inbreeding rate among all repetitions, which was 8.50%. Relative to the average inbreeding rates, the standard deviations amounted to between 25.6% and 34.9%.

## Discussion

### Genetic parameters

Due to polyandry and the fact that traits are influenced by queen and workers, the theory of heritabilities for honeybees is more involved than for most other species. In general, one distinguishes between the maternal heritability $h_m^2$, the direct heritability $h_d^2$ and the total heritability $h^2$. These can be calculated via the formulas [69, 72]

$$\begin{aligned} h_m^2=\frac{\sigma _{A,m}^2}{\sigma _{A,m}^2+\sigma _{A,md}+a_{ww}\sigma _{A,d}^2+\sigma _E^2}, \end{aligned}$$

$$\begin{aligned} h_d^2=\frac{\sigma _{A,d}^2}{\sigma _{A,m}^2+\sigma _{A,md}+a_{ww}\sigma _{A,d}^2+\sigma _E^2}, \end{aligned}$$

and

$$\begin{aligned} h^2=\frac{\sigma _{A,m}^2+\sigma _{A,d}^2+2\sigma _{A,md}}{\sigma _{A,m}^2+\sigma _{A,md}+a_{ww}\sigma _{A,d}^2+\sigma _E^2}, \end{aligned}$$

where $a_{ww}$ denotes the average relationship between workers in a colony. The occurence of $a_{ww}$ in these formulas implies that heritabilities depend on the mating system. In particular, as noted already by Kistler et al. [59], the same set of genetic parameters leads to slightly different heritabilities when queens are inseminated with sperm from different numbers of drones. The heritabilities for the simulated traits were as follows. For $r_{md}=-0.18$, the maternal heritability ranged from $h_m^2=0.16$ ($N_D=1$) to $h_m^2=0.17$ ($N_D=50$), the direct heritability ranged from $h_d^2=0.32$ ($N_D=1$) to $h_d^2=0.35$ ($N_D=50$) and the total heritability was between $h^2=0.40$ ($N_D=1$) and $h^2=0.43$ ($N_D=50$). For the stronger negative correlation, $r_{md}=-0.53$, we had $h_m^2\in [0.17,0.19]$, $h_d^2\in [0.35,0.38]$, and $h^2\in [0.26,0.29]$. These values fall well into the range of estimated heritabilities for economically relevant traits, such as honey yield or gentleness [24, 65, 73]. In particular, regularly observed phenomena like a negative correlation between maternal and direct effects or a higher direct genetic variance than maternal genetic variance are reflected by the chosen parameters.

The influence of $r_{md}$ on genetic progress and inbreeding was in line with results from the literature. Stronger negative values of $r_{md}$ diminish the total heritability of the trait, resulting in slower genetic progress [47, 59, 61, 62]. Furthermore, under BLUP selection, a reduced total heritability implies that the importance of a colony’s own performance is diminished in comparison to the performances of close relatives [74, 75]. This explains the increase in inbreeding rates for $r_{md}$. An earlier study, in which a wide range of genetic parameters was simulated [76], strongly suggests that the dependence of inbreeding development and genetic gain on the total heritability of the trait would likely also be seen if we had chosen different genetic parameters.

### Influences of $N_Q$ and $N_S$

Altering the numbers $N_Q$ and $N_S$ of selected dams and sires, respectively, are the main options for the breeder to determine the intensity of selection [71]. While a sister group size of 5 queens (corresponds to $N_Q=100$) and a large number of sires (i. e. $N_S=80$) has been found as theoretically ideal for sustainable breeding of populations with 500 colonies per year [71], sharper selection (represented by $N_Q=50$, $N_S=40$) is often practiced in reality [59, 60, 73]. Evidently, a sharper selection decreases the effective population size and leads to faster genetic progress and over the relatively short time-span of 20 years, no detrimental effects due to a depletion of genetic variance are to be expected [62, 77]. The observation that the number of dams hereby plays a greater role than the number of sires is remarkable because our values for $N_Q$ (50 and 100) were slightly larger than those for $N_S$ (40 and 80). According to general theory, effective population sizes considering different sexes are dominated by the sex with fewer reproducing individuals [78]. We assume that this effect is founded in the honeybee’s polyandry and haplodiploid genetics but have no straightforward explanation for the phenomenon.

The combination of $r_{md}=-0.18$, $N_Q=100$, and $N_S=80$ was the only setting for which generational inbreeding rates were below 1% and for which the breeding schemes were thus sustainable by standards of the Food and Agriculture Organization of the United Nations (FAO) [79]. However, it was not the aim of this study to find sustainable breeding schemes but to show the influence of the number of drones $N_D$ per insemination on breeding results. Nevertheless, it is worth to note what the barplots in Figs. 1 and 2 imply: Even if a breeding scheme is sustainable on average, a concrete realization of the scheme, i. e. a real-world breeding program, can easily produce non-sustainable results. Thus, whenever honeybee breeding is performed in reality, a close monitoring of the actual inbreeding development is highly advisable.

### Influence of $N_D$

The influence of the number $N_D$ of drones per inseminated queen on honeybee breeding results was the main focus of our study. Figures 1 and 2 reveal that all eight combinations of accompanying parameters $r_{md}$, $N_Q$, and $N_S$ yielded parallel curves for the breeding outcomes dependent on $N_D$. We thus conclude, that the main observations discussed below will also hold for any other realistic set of genetic parameters and selection intensities.

Our results showed a clear distinction between SDI and MDI strategies. In general, by the use of SDI it was possible to enhance genetic gain at the cost of higher inbreeding rates. This is in contrast to previous simulation results by Kistler et al. [59], where SDI had not paid off in terms of genetic progress. The difference between the study of Kistler et al. [59] and the present study is that we based our selection decisions on BLUP-estimated breeding values instead of mere phenotypic records. Thus, the genetic evaluation benefited from the more accurate relationship matrix under SDI leading to more accurate selection decisions.

Comparing MDI breeding schemes with different numbers $N_D$ of drones per insemination showed little to no differences in terms of genetic progress and inbreeding. Only due to the high number of repetitions, some of the differences for small numbers of $N_D$ were statistically significant with the trend that higher levels of polyandry went with slightly lower genetic progress and slightly lower inbreeding rates. It seems likely that by further increasing the number of repetitions per setting, these trends could be corroborated. The key to the explanation of the observed effects lies in the variance of paternal genetic contributions. In SDI, all female offspring of a queen inherit the same paternal genetic information. Only in MDI, there is variance among the paternally inherited alleles of siblings. This variance increases with the number of drones involved in the mating process but the marginal benefits of further drones decrease with the number of drones.

### Practical implications

Our simulations suggest that SDI can indeed constitute an interesting breeding strategy for honeybees. In particular, when one is interested in fast genetic progress over few generations, it appears as a promising strategy. Propagations of this strategy, such as e. g. by Harbo [31], are therefore not unfounded. However, when practically applying SDI, one has to cope with the fact that the queen’s spermatheca is insufficiently filled and the queen will likely only live for several months. When SDI and MDI colonies are evaluated alongside each other, this is likely to cause non-genetic structural differences in their performances, leading to biased breeding value estimations. This effect can be eliminated or at least alleviated by introducing a fixed effect (SDI or MDI) to the evaluation model [33]. Taking these considerations into account, we judge, that SDI should only be used in specific set-ups and is likely to have its main application in scientific contexts. For decentrally organzied and sustainable honeybee breeding endeavors, that rely on the inclusion of many breeders, it is important that the breeding scheme is easily manageable [26, 80, 81]. This will rather be the case for MDI or mating on isolated mating stations.

In contrast, the possibilities to optimize MDI breeding schemes via the number of drones per insemination appeared slim. Although in some cases we observed significant differences for different small values of $N_D$, one has to keep in mind that when sample sizes are big, statistical significance alone is no reliable indicator for biological relevance [82]. Instead, measures for the effect size have to be considered. Here, the observed differences were generally small. Thus, in comparison to other factors affecting the breeding outcomes, such as mating control [15], selection intensity [71], assumed genetic parameters [76], or the possible inclusion of genomic information [83–85], the effect of the number of drones will be negligible. Previous simulation studies have shown that rather than the mere number of drones per mating, the genetic variability of the involved drones can have a strong effect [47, 61, 68, 86]. Another argument that speaks against trying to optimize MDI breeding schemes for honeybees with small numbers $N_D$ of drones per insemination is that it combines the bad elements of SDI and MDI: While the genetic progress is significantly lower than in SDI, the spermatheca will still be insufficiently filled.

On the other side of the spectrum of possibilities, single colony inseminations with more than 20 drones are rarely performed because in these cases not all of the collected sperm can be used. Our simulations clearly indicate that such a practice would not provide any theoretical benefits and should therefore be abstained from.

As a consequence, we do not see any urge to change the currently common practice to focus on collecting a sufficient amount of sperm per insemination without putting much attention to the number of drones this requires.

## Data Availability

The datasets used and/or analysed during the current study are available from the corresponding author on reasonable request.
